# A case of chronic retinal necrosis after tube shunt surgery for secondary glaucoma associated with cytomegalovirus corneal endotheliitis

**DOI:** 10.1186/s12886-021-02019-w

**Published:** 2021-06-10

**Authors:** Katsue Imamachi, Aika Tsutsui, Kaoru Manabe, Masaki Tanito

**Affiliations:** 1 Department of Ophthalmology, Shimane University Faculty of Medicine, 89-1 Enya-cho, Izumo, Shimane 693-8501 Japan; 2 Devision of Ophthalmology, Matsue Red Cross Hospital, Matsue, Shimane Japan

**Keywords:** Chronic retinal necrosis, Cytomegalovirus corneal endotheliitis, Polymerase chain reaction, Tube shunt surgery, Case report

## Abstract

**Background:**

We report a case of chronic retinal necrosis (CRN) combined with cytomegalovirus (CMV) corneal endotheliitis.

**Case presentation:**

An 80-year old man was diagnosed with CRN that developed after tube shunt surgery with vitrectomy for secondary glaucoma associated with CMV corneal endotheliitis. After the use of oral valganciclovir and panretinal photocoagulation, the retinal lesion resolved rapidly and he has maintained visual acuity better than before the onset of CRN.

**Conclusions:**

Use of oral valganciclovir, prophylactic panretinal photocoagulation for the non- perfusion area and vitrectomy were effective in maintaining the visual acuity for the patient with CRN.

## Background

CRN, a new disease that was first described in 2013, is a slowly progressive occlusive vasculitis and granular retinitis in immunocompetent hosts. Its association with CMV-related inflammation is suspected [1]. We present a case of CRN that developed after implantation of an Ahmed Glaucoma Valve (New World Medical, Rancho Cucamonga, CA) with vitrectomy for secondary glaucoma associated with CMV corneal endotheliitis. Most previous cases of CRN had poor visual outcomes due to the complications, our case maintained visual acuity better than before the onset of CRN after the use of oral valganciclovir and prophylactic panretinal photocoagulation for the non- perfusion area. CRN combined with CMV endotheliitis has not been reported previously.

## Case presentation

**Fig. 1:**
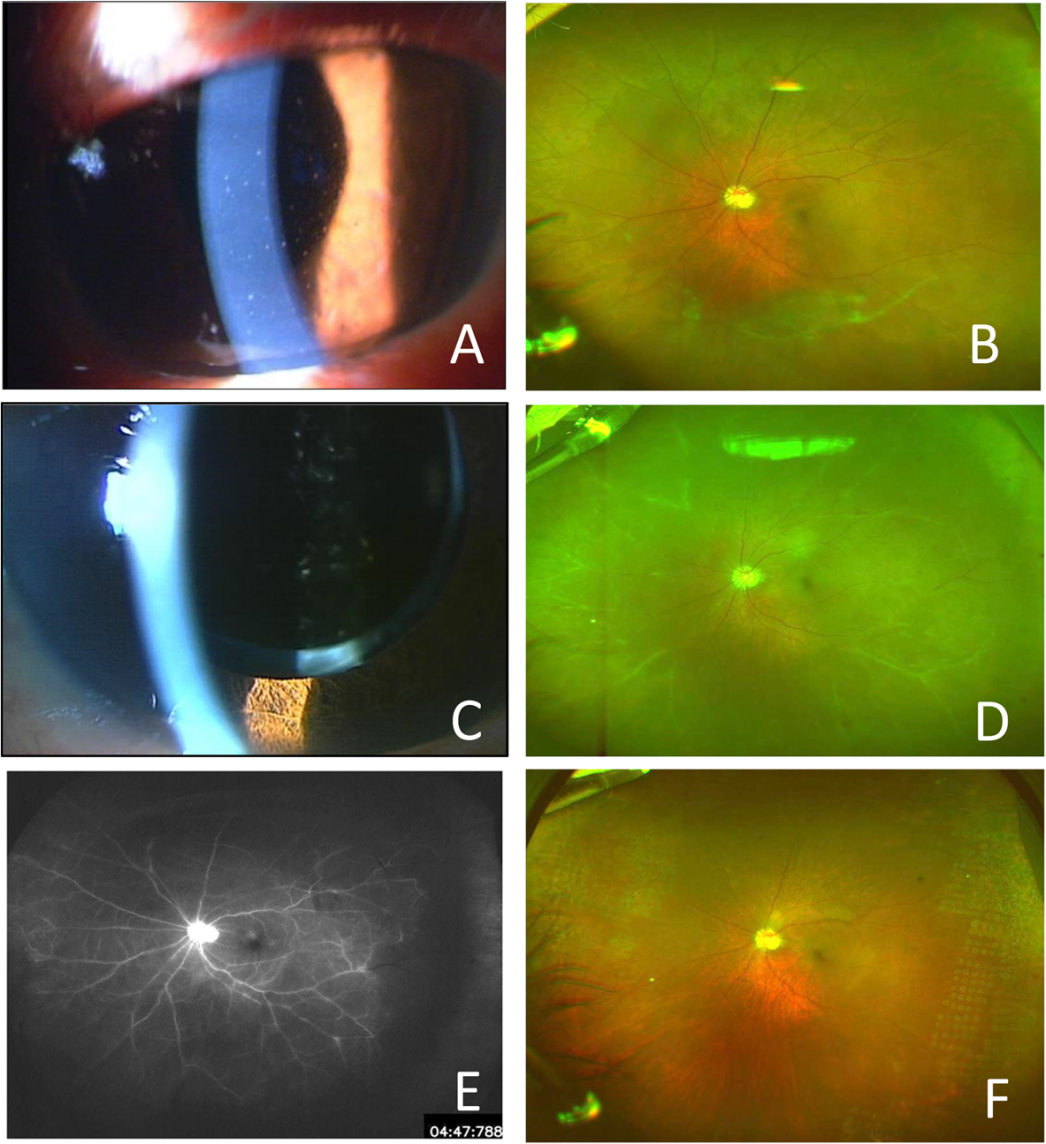
Initial findings (**A**, **B**), after topical ganciclovir (**C**), at the onset of chronic retinal necrosis (**D**, **E**), and at the final visit (**F**). At the initial visit, slit-lamp examination shows keratic precipitates and anterior chamber cells in the left eye (OS) (**A**). A wide-field fundus camera photograph shows no retinitis or occlusive retinal vasculitis OS (**B**). Two weeks after the start of ganciclovir and steroid therapy, the iritis has resolved (**C**). Two months after the glaucoma surgery (3 months after the initial visit), occlusive vasculitis in the entire fundus and granular white lesions in the nasal fundus are seen OS (**D**); fluorescence angiography shows a non-perfusion area extending throughout the fundus (**E**). After combined therapy of oral ganciclovir for 3 weeks and panretinal photocoagulation, the occlusive vasculitis and granular retinitis have resolved (**F**)

Our case is an 80-year-old man referred to our hospital with iritis and poor intraocular pressure (IOP) control in his left eye (OS). At the initial visit, the best-corrected visual acuity (BCVA) was 1.2 in the right eye (OD) and 0.3 OS, and the IOPs were 13 and 58 mmHg, respectively. Other than anterior chamber inflammation (Fig. 1a) and glaucoma, the fundus was normal (Fig. 1b). The patient had an ocular history of small-incisional cataract surgery and intraocular lens implantation OS 1 year previously. Except for systemic hypertension, he had no systemic diseases associated with immune deficiency and his human immunodeficiency virus (HIV) testing result was negative. The diagnosis of CMV corneal endotheliitis OS was made based on the previously-reported diagnostic criteria [2]. Detection of 5.2 × 10^5^ copies/ml CMV DNA in the aqueous humor from the affected eye by a polymerase chain reaction (PCR) assay and keratic precipitates like coin-shaped lesion were observed. Serum IgM of Herpes simplex virus (HSV), Varicella zoster virus (VZV), and CMV were negative, and these IgG titers were slightly elevated to 61.8, 13.0, and 15.8, respectively; the CMV antigenemia was negative. After starting the treatment (0.5% ganciclovir eyedrops 6 times/day and 0.1% betamethasone 4 times/day) with the previously reported regimen [3], the anterior-segment inflammation resolved (Fig. 1c). Because the IOP control was poor despite four ocular antihypertensive drugs, tube shunt surgery using the Ahmed Glaucoma Valve (Model FP-7) was implanted 1 month after the initial visit. To preserve the corneal endothelial cells, a tube was inserted into the vitreous cavity [4]; for this purpose, 25-gauge pars plana vitrectomy was performed intraoperatively. After the glaucoma surgery, the IOP decreased below 10 mmHg with continuous use of topical ganciclovir and betamethasone without ocular antihypertensive medications. At the follow-up visit 2 months postoperatively, although he was unaware of visual worsening, granular retinitis and occlusive vasculitis were observed OS (Fig. 1d). At this time, PCR identified 1.8 × 10^4^ copies/ml CMV in the aqueous humor. CRN associated with CMV infection was suspected, and oral valganciclovir (1800 mg for 2 weeks and then 900 mg for 1 week) was started in addition to the topical ganciclovir and betamethasone. Since the non-perfusion area extended throughout the entire fundus (Fig. 1e), panretinal photocoagulation was performed simultaneously. After valganciclovir was started, the retinal lesions resolved rapidly (Fig. 1f). At the final visit 12 months after the diagnosis of CRN, the BCVA and IOP were 0.6 and 11 mmHg, respectively, and the number of corneal endothelial cells of 2031 cells/mm^2^ before the tube shunt implantation in the vitreous was maintained 2038 cells/mm [2]. The inflammation did not recur in the anterior segment and fundus during the follow-up period with a maintenance dose of topical 0.5% ganciclovir 4 times/day and 0.1% fluorometholone 4 times/day. Iris or angle neovascularization was not seen during the follow-up period.

## Discussion and conclusions

In the current case, the fundus lesion developed after tube shunt glaucoma surgery combined with vitrectomy in the pseudophakic eye with CMV endotheliitis and iritis, and CMV DNA in the aqueous humor was detected by PCR assay before and after the onset of CRN. Thus, the procedure might have facilitated transition of CMV virus from the anterior segment to the fundus, although this speculation required to be proved. CRN combined with CMV endotheliitis has not been reported previously. To the best of our knowledge, three reports of seven CRN cases have been published [1, 5, 6]. In the initial report of five CRN cases [1], a retinal detachment developed in one case and neovascular complications developed in four cases during the follow-up. Another case of CRN complicated by severe neovascular glaucoma was reported in Japan [5]. Although most previous cases had poor visual outcomes due to complications, our patient has maintained visual acuity more than before the onset of CRN. Use of oral valganciclovir, prophylactic panretinal photocoagulation and vitrectomy may explain the maintaining of visual acuity in the current case.

## Data Availability

All data generated during this study are included in this published article.

## Abbreviations

CRN: Chronic Retinal Necrosis
CMV: Cytomegalovirus
IOP: Intraocular pressure
BCVA: Best-corrected visual acuity
HIV: Human immunodeficiency virus
PCR: Polymerase chain reaction
